# Optimized workflow of site-specific cryo-lamella preparation for cryo-ET using integrated light and electron microscope

**DOI:** 10.52601/bpr.2025.250016

**Published:** 2026-08-31

**Authors:** Weixing Li, Ke Xiao, Jiali Xie, Maoge Zhou, Yuanyuan Li, Wei Ji

**Affiliations:** 1 Institute of Biophysics, Chinese Academy of Sciences, Beijing 100101, China; 2 Bioland Laboratory, Guangzhou 510005, China; 3 Guangzhou National Laboratory, Guangzhou 510005, China; 4 College of Life Science, University of Chinese Academy of Sciences, Beijing 100049, China

**Keywords:** Cryo-electron tomography (cryo-ET), Focused ion beam (FIB), Correlative light and electron microscopy (CLEM), Integrated cryo-CLEM workflows

## Abstract

Cryo-electron tomography (cryo-ET) is an essential tool for resolving cellular structures in their native state. However, achieving site-specific sample preparation remains a significant challenge, particularly for deeply buried or rare cellular targets. Focused ion beam (FIB) milling is commonly used to prepare thin lamellae from vitrified samples, but traditional FIB methods often lack the abilities to target specific regions of interest. Correlative light and electron microscopy (CLEM) overcomes this limitation by combining light microscopy (LM) with scanning electron microscopy (SEM), enabling the identification and localization of structures of interest within the specimen. This targeted approach enhances the accuracy and efficiency of FIB milling by ensuring that lamellae are thinned at precisely the right locations. Recent advances in integrated cryo-CLEM workflows have streamlined this process, offering enhanced precision and reproducibility in sample preparation for cryo-ET. Here, we present an optimized protocol that utilizes this integrated approach to identify and target specific cellular structures, such as the contact sites between lipid droplets (LD) and mitochondria. This protocol facilitates the precise preparation of cryo-lamellae and enhances the efficiency of data acquisition in cryo-ET, offering a promising strategy for high-resolution structural biology studies.

## INTRODUCTION

Resolving cellular structures in situ is crucial for understanding biological processes within their native environments (Oikonomou and Jensen 2017; Strack 2020). Cryo-electron tomography (cryo-ET) is a pivotal technique for resolving cellular structures in their native state, offering insights into the intricate architecture of cells without the need for chemical fixation or dehydration (Doerr 2017; Oikonomou and Jensen 2017; Turk and Baumeister 2020). Focused ion beam (FIB) milling is a critical technique for preparing thin lamellae from vitrified biological specimens, enabling high-resolution cryo-electron tomography (cryo-ET) of cellular structures in their native state (Marko *et al.* 2007; Miroslava Schaffe and Baumeister 2015; Villa *et al.* 2013). However, conventional FIB milling has limitations in achieving site-specific sample preparation, particularly for deeply buried or rare cellular targets. Correlative light and electron microscopy (CLEM) overcomes these limitations and enables precise site-specific milling of cellular structures (Arnold *et al.* 2016; Kuba *et al.* 2021). CLEM combines light microscopy (LM) with scanning electron microscopy (SEM), allowing researchers to identify and localize specific structures of interest within vitrified cells using fluorescence markers. This targeted approach ensures that the FIB milling process precisely thins the lamella in regions containing relevant cellular components.

Using an integrated LM-FIB-SEM system to prepare lamellae for cryo-ET at specific sites is increasingly becoming a trend in structural biology (Pierson *et al.* 2024). This approach reduces risks such as ice contamination, sample devitrification *etc*. by keeping all imaging and milling steps within a single system, and it also improves the efficiency and accuracy of the CLEM experiment. However, due to the complexity of the workflow, standardization has not yet been achieved, making the generalization and widespread adoption of this method challenging. We have recently developed an optimized protocol to fully leverage the capabilities of our custom-designed LM-FIB-SEM system, known as the Correlative Light and Ion Microscope (CLIEM). The CLIEM system offers several unique advantages over existing commercial instruments, such as the Aquilos 2 with iFLM. First, CLIEM features an integrated confocal microscope capable of true 3D multicolor imaging of biological samples. Second, the correlation between EM and LM images is based on a novel “projection” principle, enabling nanometer-level image colocalization accuracy. Third, unlike conventional methods that rely on additional fiducial markers, CLIEM employs FIB-etched benchmarks as reference points for the correlation process. Using the CLIEM system and this protocol, we identified the contact sites between the lipid droplets (LD) and the mitochondria and prepared cryo-lamella exactly containing these contact sites. Moreover, we also employ the LM image of the final lamella to quickly locate the target in the transmission electron microscopy (TEM) image, facilitating the determination of the data acquisition area in cryo-ET.

## MATERIALS

### Biological materials

• HepG2 cell

### Regents

• Dulbecco’s modified Eagle’s medium (DMEM, Gibco, Cat. No. c11995500BT)

• 10% fetal bovine serum (FBS, Gibco, Cat. No. 16000-044)

• 1% penicillin-streptomycin ((10,000 U/mL penicillin and 10 mg/mL streptomycin, HyClone, Cat. No. 30010)

• MitoTracker Deep Red (M22426, Thermo Fisher Scientific)

### Software and algorithms

• Fiji/Imagej, v.1.53f51

### Equipment

• Plunge freezer: Leica EM GP 2

• Objective: MPLFLN×100/NA 0.9, Olympus

• Integrated LM-FIB-SEM system “CLIEM”

• 300 kV Titan Krios transmission electron microscope, ThermoFisher

### Other materials

• EM grid: T10012Au, Beijing XXBR Technology Co., Ltd

• Tweezers: EP5081, EP5083, EP5086, PELCO

• Autogrids: 1131750 (C-CLIP), 1188953(C-CLIP Ring), Thermo Fisher Scientific

• Sample box: LT-TEM4-2 for EM grids, LT-TEM4-4 for Autogrids, Beijing EBO Technology Limited

• Confocal dish: D35-20-1-N, Cellvis.

• Filter paper: 10311807, Whatman

## PROCEDURE

### Cell preparation

#### Stable cell line construction

1　Amplify the sfGFP1-10-v5 segment from the plx304-sfGFP1-10-Ert plasmid for the construction of the pCDH-sfGFP1-10-v5-Plin2 plasmid.

2　Clone the overlapping PCR products of sfGFP1-10- v5-plin2 into the pCDH-CMV-MCS-EF1-Puro vector.

3　Insert the Mito-myc-sfGFP11 sequence into the pCDH-CMV-MCS-EF1-Puro vector. The N-terminal 33 amino acids of human TOMM20 were utilized as the mitochondria-targeting signal. The Mito-myc-sfGFP11 sequence was commercially synthesized by GENEWIZ Biotechnology Co., Ltd. (Suzhou, China).

4　Co-transfect the packaging plasmids pLP1, pLP2, and pLP/VSVG into HEK293T cells together to generate the lentiviral particles stably expressing mito-sfGFP11 and sfGFP1-10-Plin2. Harvest and concentrate the lentivirus-containing supernatant after a 72-h incubation.

5　Infect the HepG2 cells with the two concentrated lentiviruses in the presence of 10 μg/mL polybrene to enhance infection efficiency. Subsequently, the infected cells were subjected to selection with 2 μg/mL puromycin.

6　Sort single GFP-positive cells via flow cytometry. Then, the positive cells are maintained in DMEM medium supplemented with puromycin.

7　Carefully select the appropriate cell clone, named the MLDCS cell line, using a confocal microscope (LSM980, Zeiss, Germany) equipped with a high-resolution 63×/1.40 NA oil-immersion objective.

**[NOTE]** Fluorescence labeling is essential for accurately locating the target under a fluorescence microscope. First, sufficient signal brightness is required for the microscope to effectively detect the fluorescence. Second, strong and consistent labeling facilitates the identification of fluorescent targets. Establishing a stable cell line is a reliable option, as it typically provides both high fluorescence intensity and labeling efficiency. Alternatively, other labeling methods, such as immunofluorescence or transient transfection, can also be used.

#### Cell culture

1　Supplement 10% (v/v) FBS, 2 μg/mL puromycin, and 1% (*v*/*v*) penicillin-streptomycin to DMEM to maintain cellular viability and prevent microbial contamination.

2　Maintain the MLDCS cell line under standard culture conditions in a humidified incubator with 5% CO_2_ at 37°C, using the DMEM above.

3　Remove the medium for the passaging of the cells (~90% confluence). Then, wash the cells with phosphate-buffered saline (PBS) to remove any residual medium and serum.

4　Add the appropriate trypsin-EDTA solution to detach the cells from the flask surface by incubating at 37°C for a short period until the cells round up and detach.

5　Add an equal volume of medium containing FBS to inactivate the trypsin.

6　Transfer the cell suspension to a centrifuge tube. After centrifuging at 1000 r/min for 5 min, the cell pellet was resuspended in fresh medium, and transferred to new culture flasks at an appropriate dilution.

#### Cell seeding on EM grids and mitochondria staining

1　Place 200 mesh lacey gold EM grids in confocal dishes.

**[NOTE]** Other grid types, such as Quantifoil grids, may also be used, provided the membrane contains holes that allow the blotting medium to be drawn away from the backside.

2　Clean the EM grids using a plasma-cleaner (Gatan Solarus 950) for 90 s with Ar and O_2_.

3　Sterilize the EM grids by UV irradiation for 30 min.

**Figure 1:**
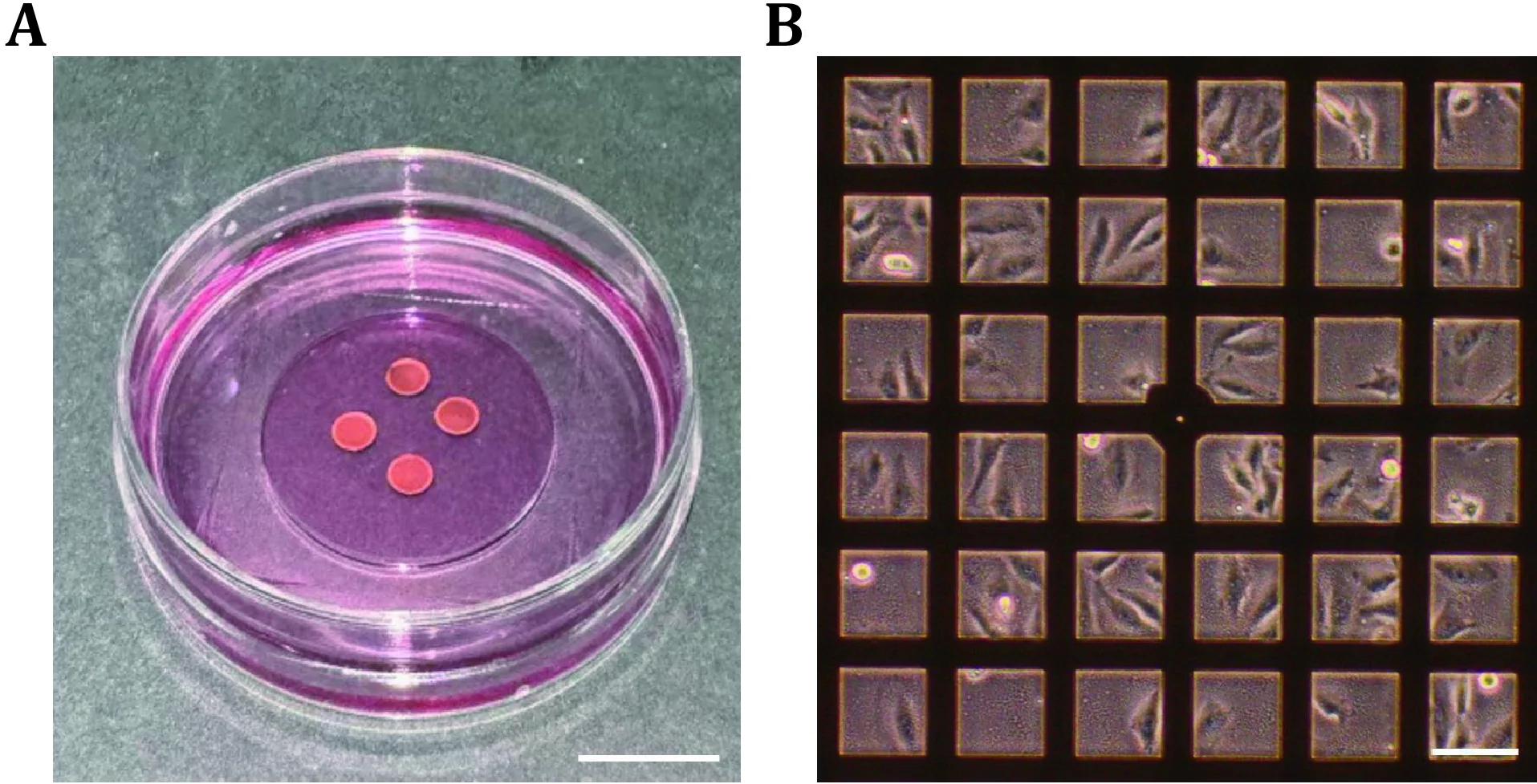
Cell seeding on EM grids. **A** Four 200 mesh lacey EM grids immersed in DMEM in a confocal dish, scale bar: 1 cm. **B** Bright field image of HepG2 cells seeded on the EM grids, scale bar: 100 μm

4　Seed the cells on the EM grids in DMEM at a density of 1 × 10^5^/mL for ~24 h to promote cell adherence and spreading (Fig. 1A). It is generally considered a good density when there are approximately 2−3 cells in each grid square (Fig. 1B).

5　Stain the cells with 1 μmol/L MitoTracker deep red.

6　Observe the fluorescent signal of the cells with a confocal microscope to make sure the cellular state is good.

### Cell vitrification

#### EM GP preparation

1　Thoroughly dry the cryogen cup and tweezers using a hair dryer for ~2 min.

2　Turn on the EM GP plunge freezer.

3　Replace the filter paper.

4　Add Milli-Q water through the rubber pipe to the EM GP.

5　Set the humidity to 85%.

6　Set the temperature to 37°C.

7　Set the temperature of the cryogen to −182°C.

8　Run the EM GP through an empty cycle to verify that the system functions correctly.

#### Cryogen preparation

1　Add liquid nitrogen to the corresponding cryogen cup of the EM GP.

2　Allow the system to cool down for 10−20 min.

3　Prepare liquid ethane in the corresponding cryogen cup of the EM GP.

#### Plunge freezing

1　Place an empty cryo-box in the corresponding cryogen cup.

2　Pick up a grid with cells from the petri dish using tweezers and lock the tweezers with the sliding block.

3　Secure the tweezers in the EM GP. Add 3 μL phosphate-buffered saline (PBS) onto the grid to dilute the cell culture medium.

**[NOTE]** The cells used in this protocol are stretched and relatively thin (less than 10 μm). In this case, the simple method of diluting the viscous cell culture medium with PBS shortly before plunge freezing already results in successful vitrification of over 80% of cells. For thicker cells, replacing the cell culture medium with cryoprotectants such as sucrose or glycerol may help to improve vitrification success.

4　Set the blot time to 3−5 s.

5　Set the post-blotting time to 0 s.

6　Set the blot type to single blot.

7　Set the blot times.

8　Start the automatic plunge freezing procedure.

#### Sample transfer and storage

1　Insert the vitrified grid into the cryo-box after plunge freezing. Ensure the grids are not exposed to air during the transfer.

2　Quickly place the cryo-box into a 50 mL centrifuge tube filled with liquid nitrogen.

3　Transfer the centrifuge tube to a liquid nitrogen tank for storage.

### Fluorescence guided FIB milling

#### Sample loading

1　Cool down the cryo-stage in the LM-FIB-SEM.

2　Take a sample from the liquid nitrogen tank.

3　Mount vitrified EM grids in Autogrids in a cryo-workstation.

4　Mount the Autogrids in the SEM sample holder.

5　Transfer the sample holder into the LM-FIB-SEM using the equipped cryo-transfer system.

#### Pt coating

1　Set the GIS temperature to 30 degrees and heat it for 15 min.

2　Outgas the GIS once or multiple times until the vacuum level stabilizes and remains above 10^-^^3^ Pa.

3　Move the sample to a position 2 mm above the FIB-SEM coincidence point.

4　Insert the GIS nozzle.

**Figure 2:**
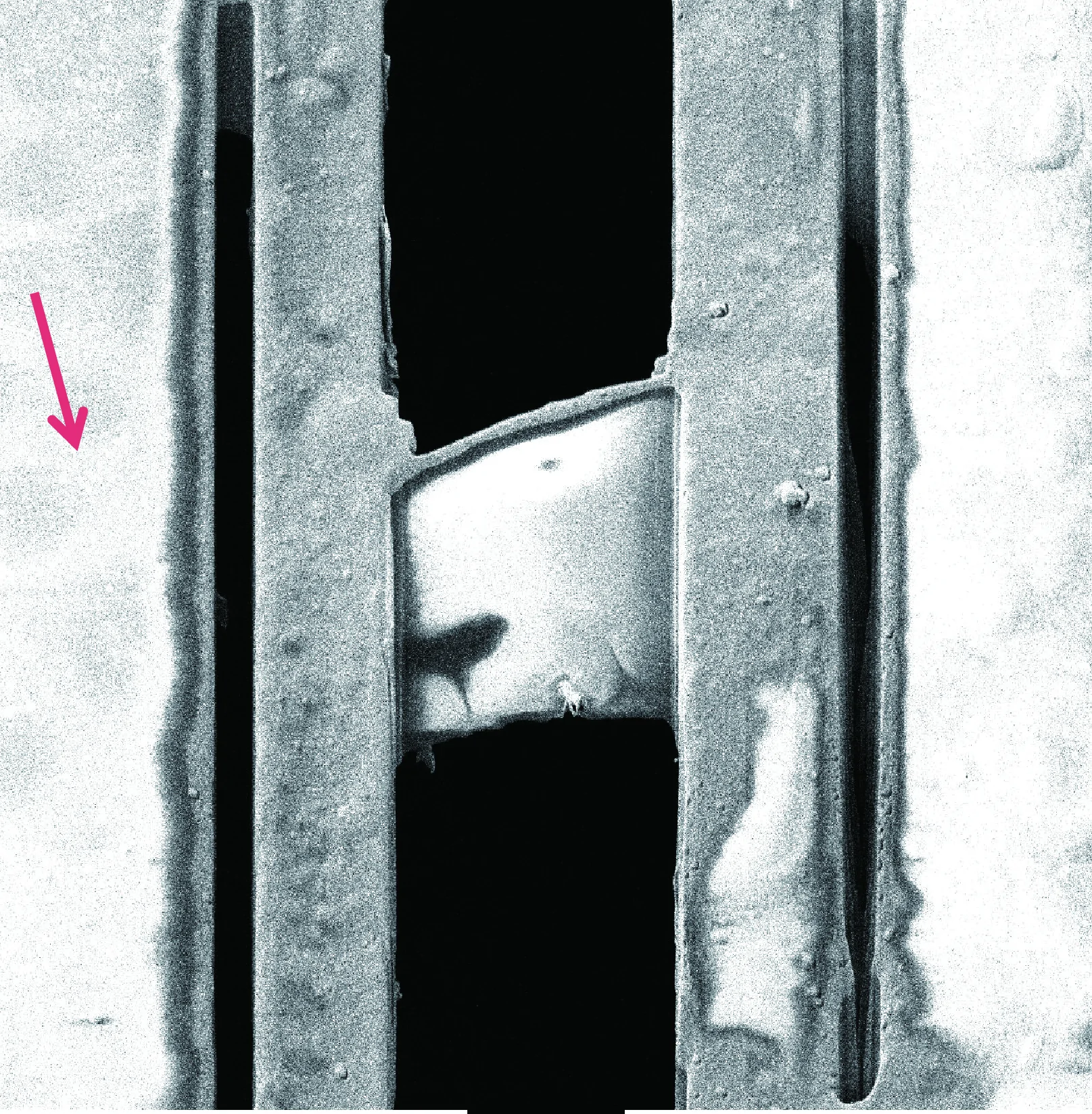
Pt layer (red arrow) observed under SEM. Scale bar: 5 μm

5　Open the GIS valve and coat for 40–60 s, coat a Pt layer (Fig. 2).

**[NOTE]** The primary purpose of the Pt coating is to protect the sample surface during the FIB milling process. A Pt layer of ~500 nm thick helps the sample withstand lamella preparation and minimizes curtain artifacts, thereby improving both milling and imaging quality. The required coating time may vary depending on the sample type. To determine the optimal duration for a specific sample, one can vary the coating time and cut a test window to measure the resulting Pt layer thickness. For vitrified single-cell samples, as used in this protocol, a coating time of 40–60 s is generally appropriate.

6　Retract the GIS to the parking position.

#### SEM imaging and cross mark milling

1　Image the cells on the EM grid using SEM at a working distance of 6 mm. Typical SEM settings are 5 keV and 30 pA.

2　Choose an appropriate target cell that is single-layer and located within the grid.

3　Tilt the stage by 17 degrees.

**Figure 3:**
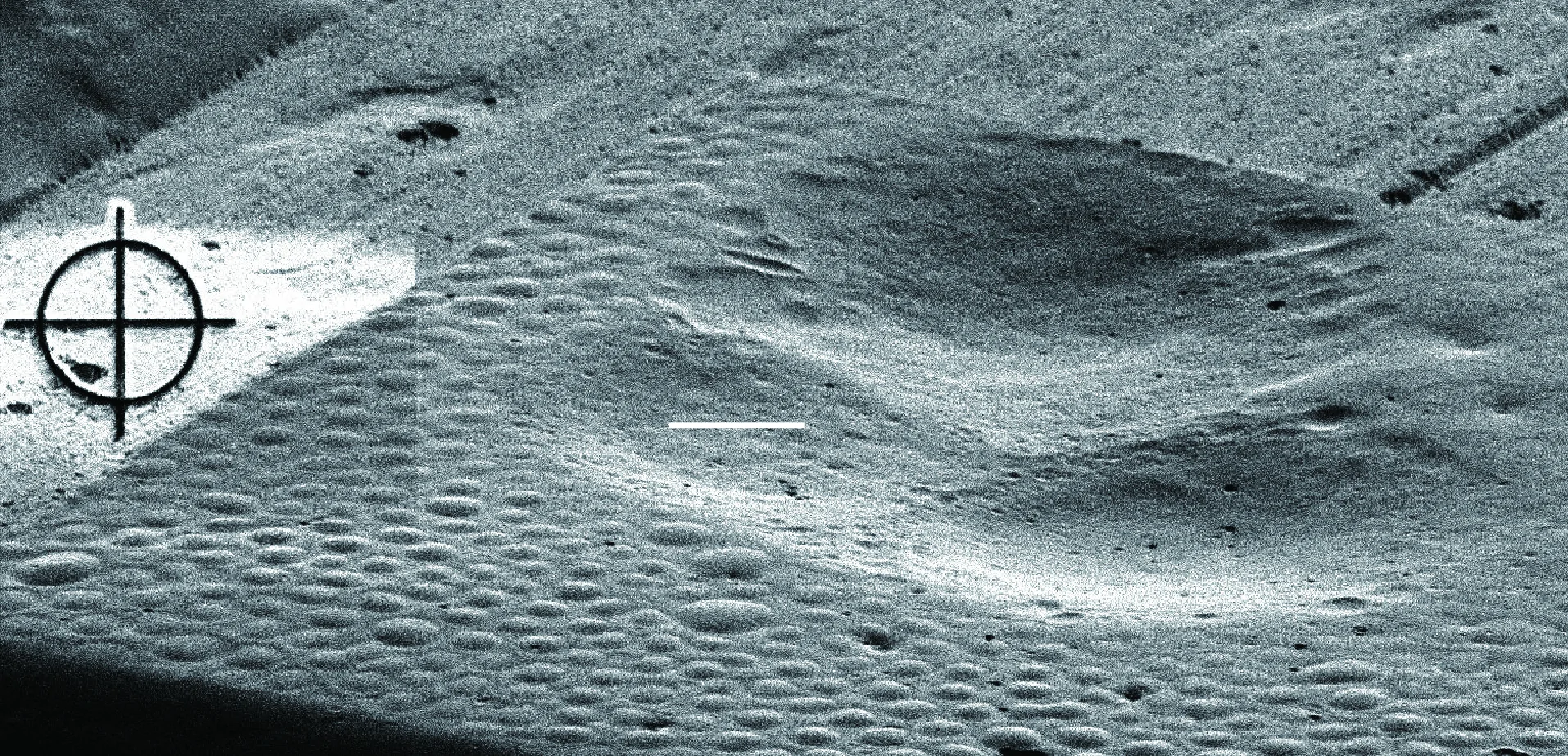
A cross mark milled on the metal bar using FIB. Scale bar: 10 μm

4　Mill a cross mark on the metal bar next to the ROI using an FIB beam current of 500 pA (Fig. 3).

#### 3D LM imaging

1　Move the stage to the corresponding LM position that captures the same ROI.

2　Image the cells using the integrated confocal microscope.

3　Choose an appropriate area that contains the interested target with a decent fluorescence signal.

**Figure 4:**
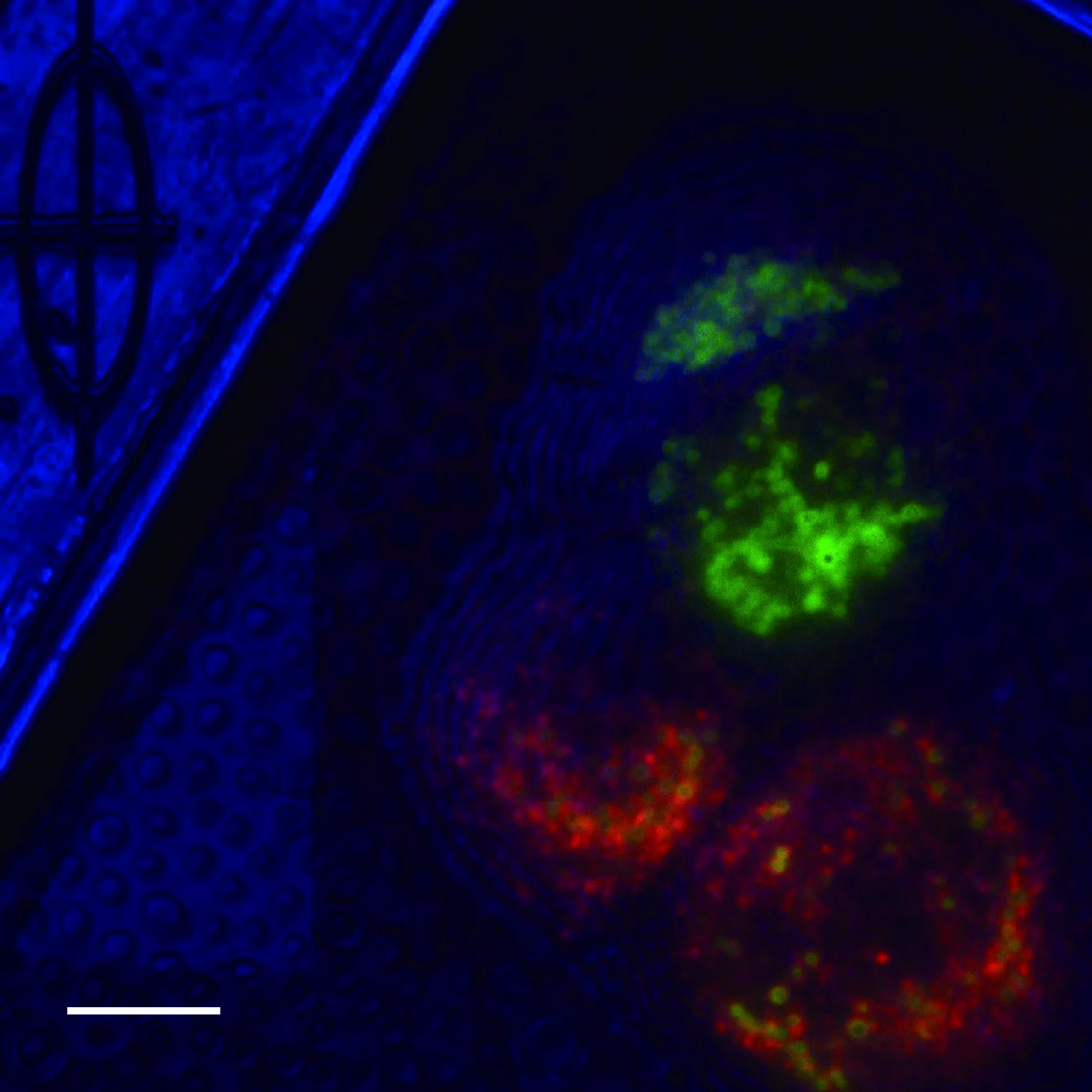
Projected 3D LM image containing three channels, showing the cross mark (bright field) and two fluorescently labeled cells (green, LD-mitochondria contact sites; red, mitochondria). Scale bar: 10 μm

4　Collect the 3D image of this region, including the cross marker, using suitable magnification. The typical z-step is 100−200 nm (Fig. 4).

#### Target localization in LM image

**Figure 5:**
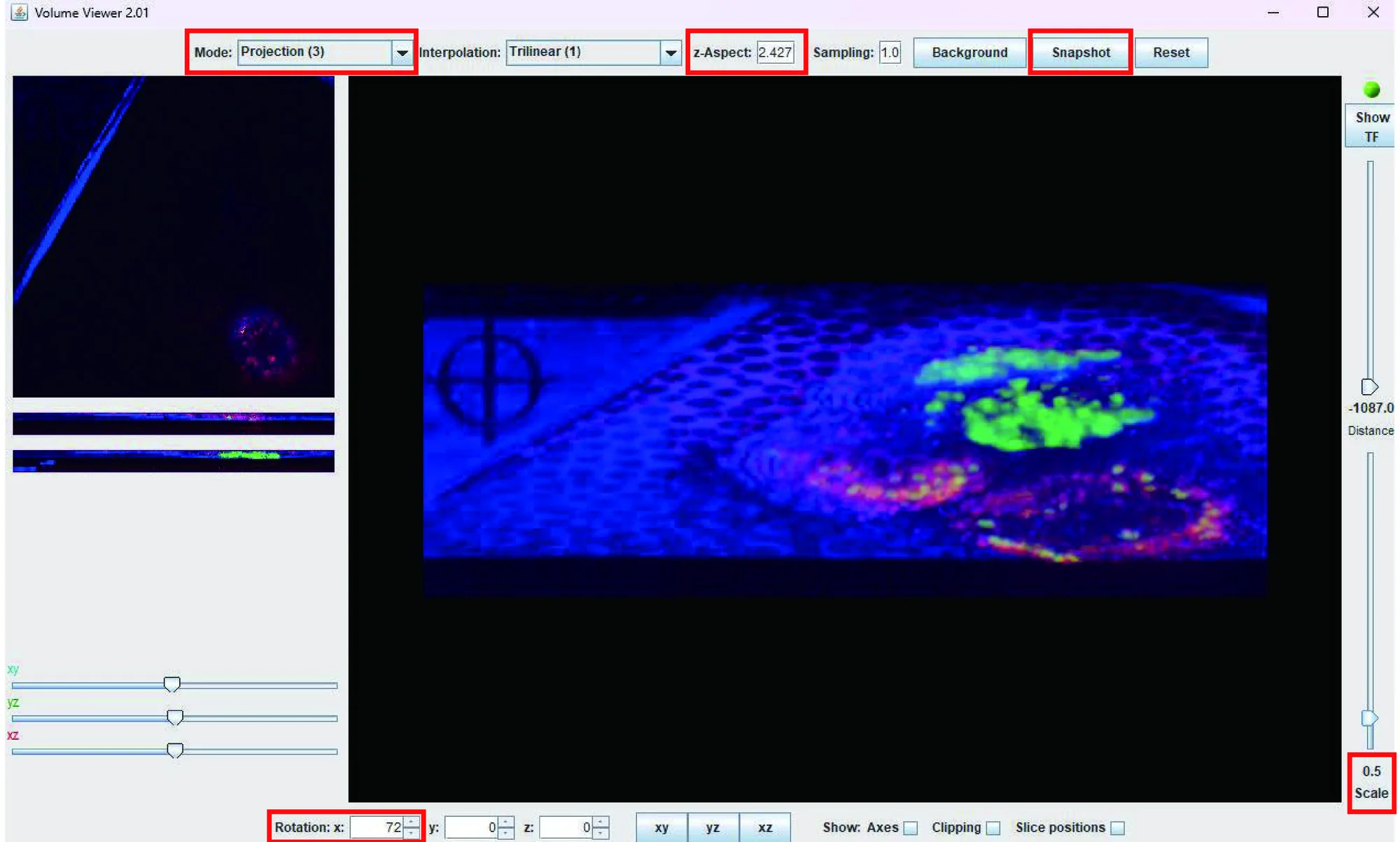
Volume Viewer interface and settings

1　Open the 3D LM image in Fiji/ImageJ, display the image in the “Volume Viewer” plugin (Fig. 5).

2　Set “mode” to “projection” and “rotation x” to 72 degrees (corresponding to the FIB milling angle). This step projects the 3D LM image onto the FIB angle, producing a 2D LM image with an identical perspective to the FIB image.

3　Set the “z-aspect” value according to the pixel size, the z-step and the image scale. For example, for the image taken at the 1536 × 1536 resolution with a magnification of 2× (corresponding pixel size 82.39 nm) and the z-step of 200 nm, the z-aspect = z-step/pixel size = 200 nm / 82.39 nm = 2.427. This step ensures the accuracy of the projection in the previous step.

**[Note]** The pixel size of the confocal image can be calibrated using various methods. For example, one can use microscopy calibration targets (*e*.*g*., R1L3S5P from Thorlabs) with patterns of known dimensions to determine pixel size. Alternatively, a pattern, such as a line of known dimensions, can be etched using the FIB and then imaged with the confocal microscope in brightfield mode. In contrast, the pixel size of the SEM/FIB image is typically a default system value that is pre-calibrated by the manufacturer.

4　Choose an appropriate scaling ratio to display the full projected image in the window.

5　Take a snapshot of the projected image by clicking the “Snapshot” button.

**Figure 6:**
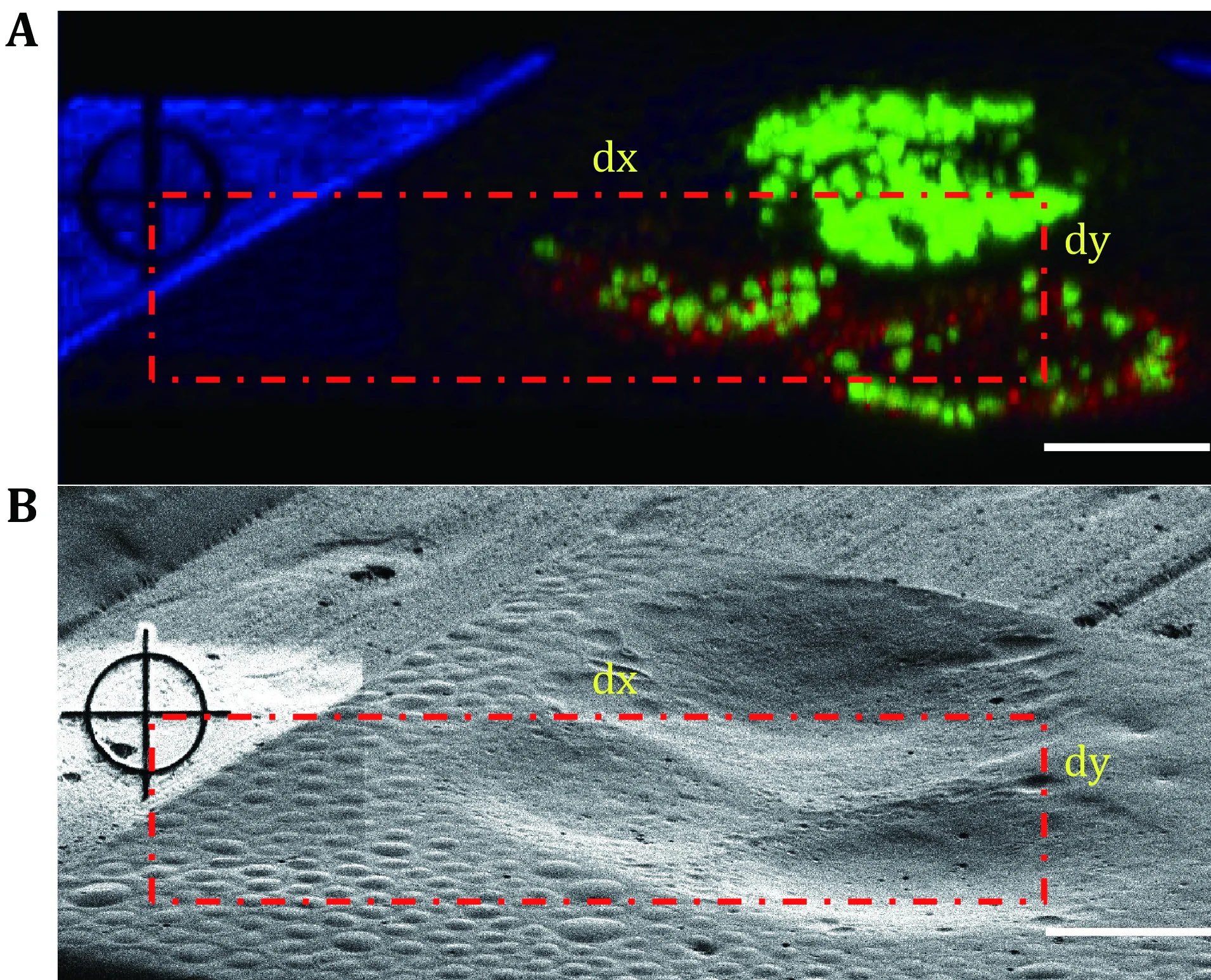
A Target localization in LM image. B Target localization in FIB image. Scale bar: 10 μm

6　Determine the target position by measuring the distance between the center of the cross mark and the center of the target in pixels on the snapshot (Fig. 6A).

7　Convert the measured pixel distances to real distances: real distances = pixel distance × pixel size / scale. These real distances will be used to determine the target position in the FIB image in the following steps.

#### Target localization in FIB image

1　Move the sample back to the SEM position.

2　Tilt the stage by 17 degrees.

3　Take an FIB image of the cells and the cross mark.

4　Draw a rectangle on the FIB image from the center of the cross mark, which has the side length of the measured x−y distances in the LM image. The target is then located at the vertex of the rectangle's diagonal (Fig. 6B).

**[NOTE]** Any mechanical drift in the imaging system can be considered negligible for distance measurements. System drift was measured at less than ~7 nm per minute. Given that the confocal image stack and FIB/SEM image acquisition times are ~2 and ~0.2 min, respectively, the corresponding drift would be less than ~14 and ~1.4 nm. These values are insignificant when compared to typical measured distances on the micrometer scale.

#### Site-specific FIB milling

1　Mill two rectangles of ~10 × 6 μm above and below the determined target position using a beam current of 500 pA, leaving a lamella of roughly 3-μm thick.

**Figure 7:**
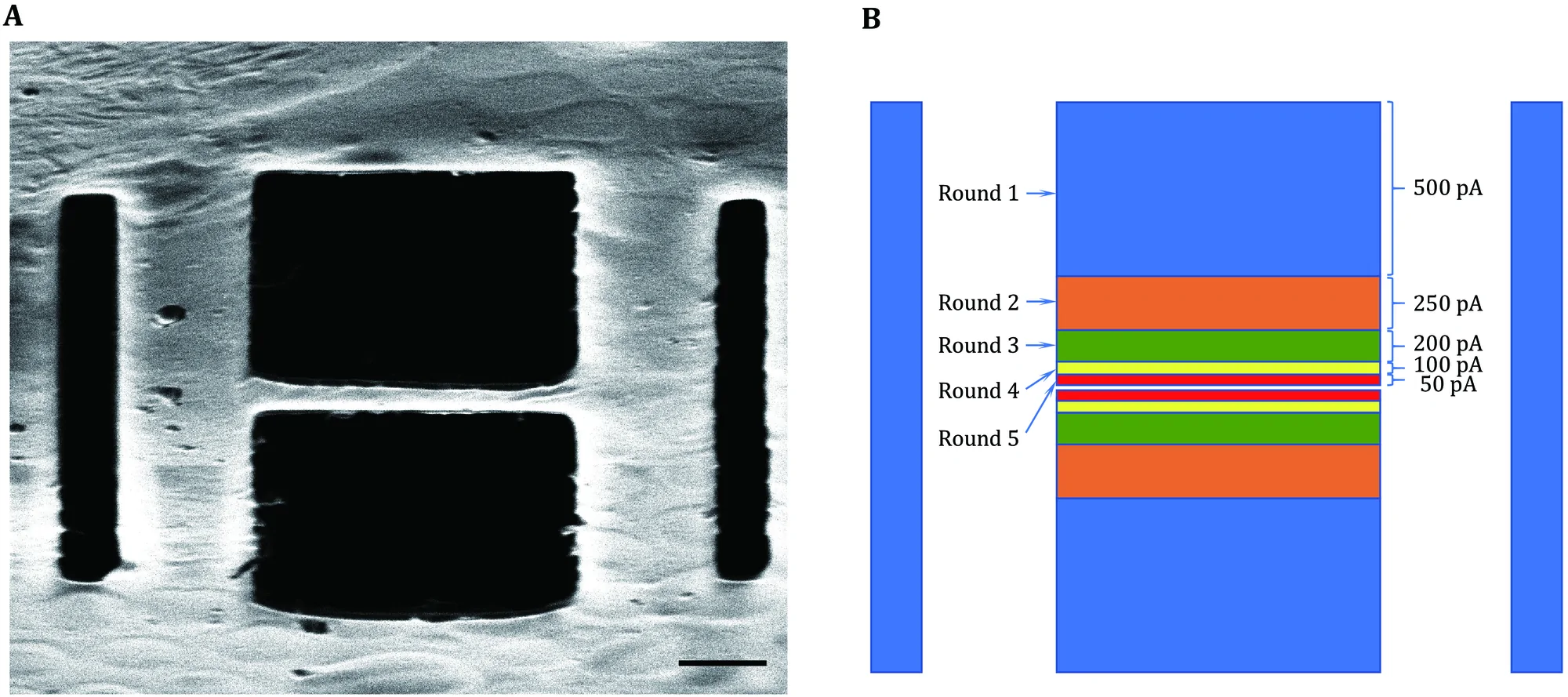
FIB milling process. **A** FIB image of an FIB-milled lamella and stress-relief trenches. **B** A typical FIB thinning process. Scale bar: 2 μm

2　Mill two stress-relief trenches of 0.5−1 μm wide on both sides of the lamella using a beam current of 500 pA (Fig. 7A).

3　Gradually reduce the beam current from 500 to 50 pA to thin the lamella to a final thickness of about 150−200 nm (Fig. 7B) using the settings in Table 1.

**Table 1:** A typical FIB thinning process and settings

Round	FIB beam current	Lamella thickness
1	500 pA	2.8−3 μm
2	250 pA	1.3−1.5 μm
3	200 pA	0.8−1 μm
4	100 pA	500 nm
5	50 pA	150−200 nm

**[NOTE]** The lamella thickness in Table 1 was measured in FIB images via standard tools in the FIB/SEM operation software.

**Figure 8:**
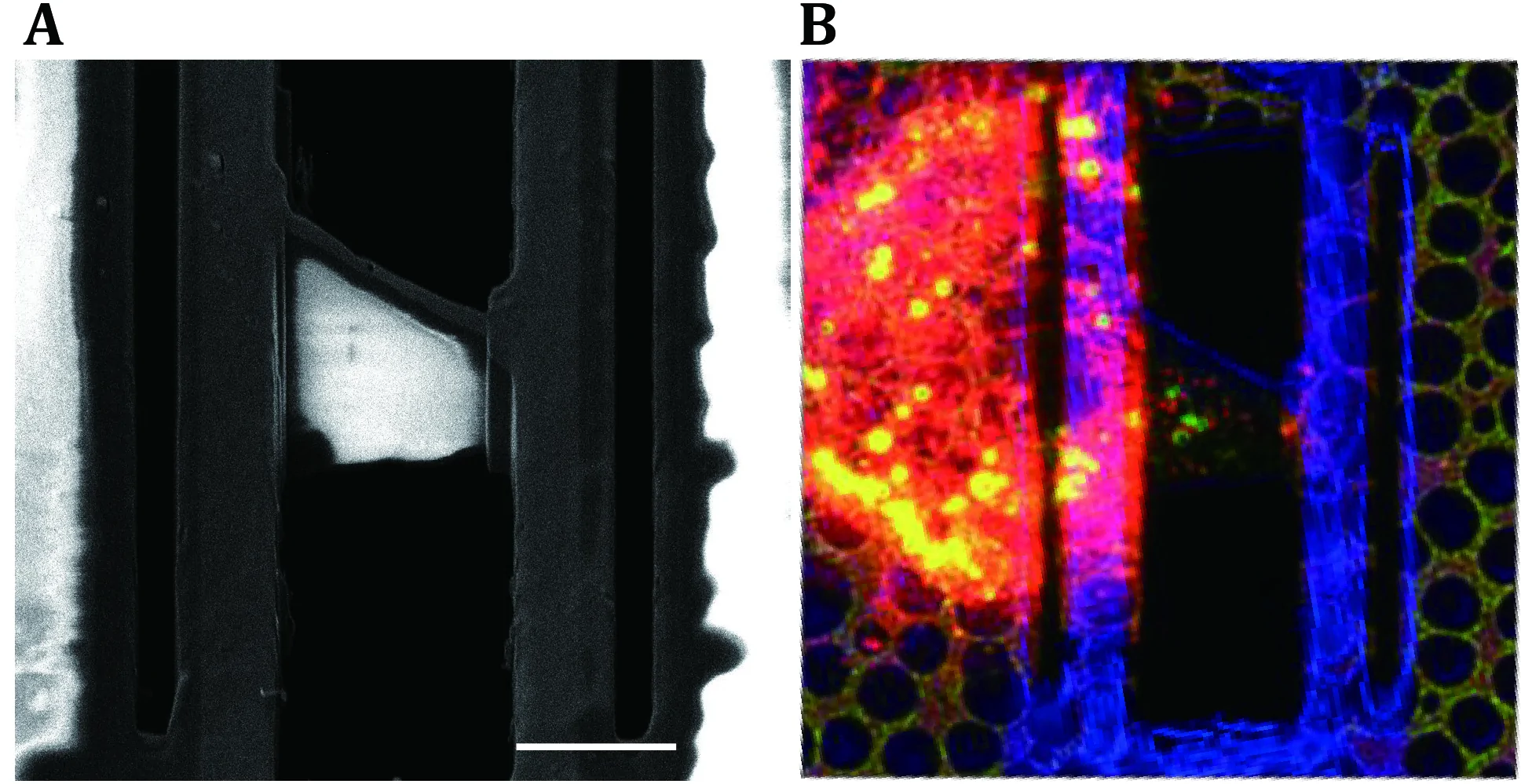
**A** The SEM image of the prepared lamella. **B** The LM image of the prepared lamella. Scale bar: 5 μm

4　One can check the fluorescence signal of the lamella at any milling step to ensure the target signal is present in the lamella (Fig. 8).

**[NOTE]** The accuracy of this site-specific milling procedure is in the order of several tens of nanometers (Li *et al.* 2023b), allowing for precise milling of subcellular organelles, viruses, macromolecular complexes, and similar structures. The overall success rate of the site-specific milling approach is typically over 95% (Li *et al.* 2023b).

### LM assisted target localization in cryo-ET

**Figure 9:**
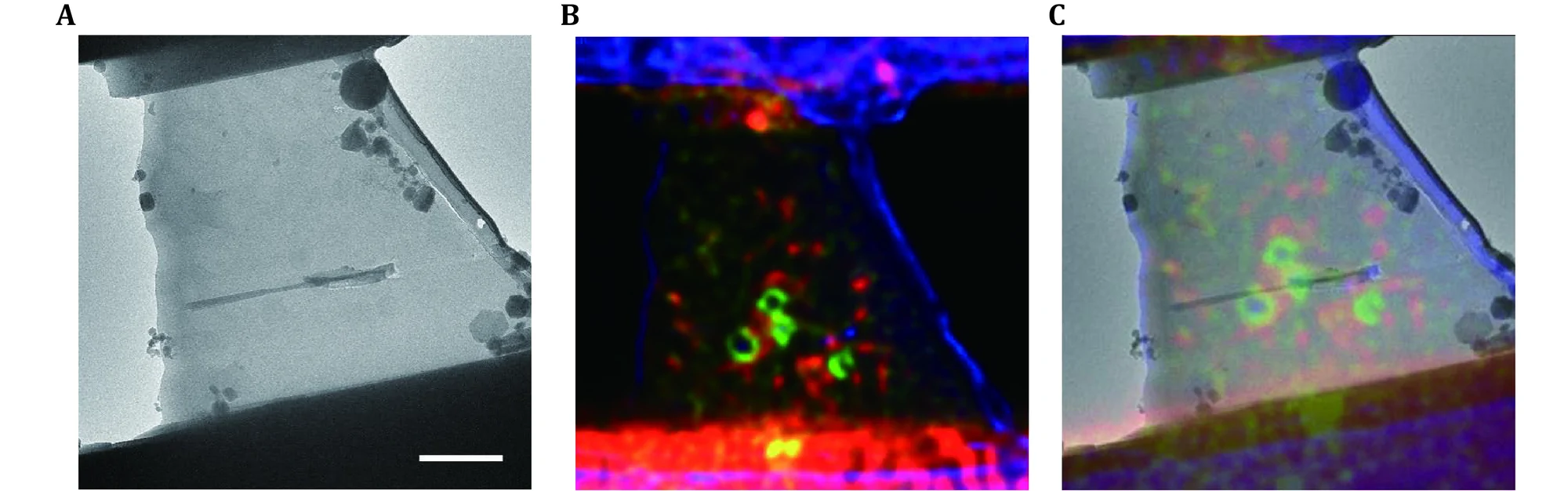
Correlation of the LM and the TEM images of the lamella. **A** TEM image of the lamella. **B** LM image of the lamella. **C** Superimposition of the LM and the TEM images. Scale bar: 2 μm

1　Scale the cryo-LM (Fig. 9A) and cryo-TEM images (Fig. 9B) of the lamella to the same magnification in accordance with the pixel size of each imaging modality.

2　Rotate the two scaled images to the same angle.

3　Superimpose the two images according to the edge information of the lamella in the bright-field channel of the LM image and the TEM image (Fig. 9C).

4　Identify the target according to the fluorescence signal.

5　Locate the target in the corresponding position in the TEM image.

### Cryo-ET data collection

1　Set the magnification to 33000X.

2　Set the tilt angle from −54 to 48 degrees with a tilt step of three degrees and the target defocus of −6 mm.

3　Set the cumulative dose to ~103 e/ Å^2^.

**Figure 10:**
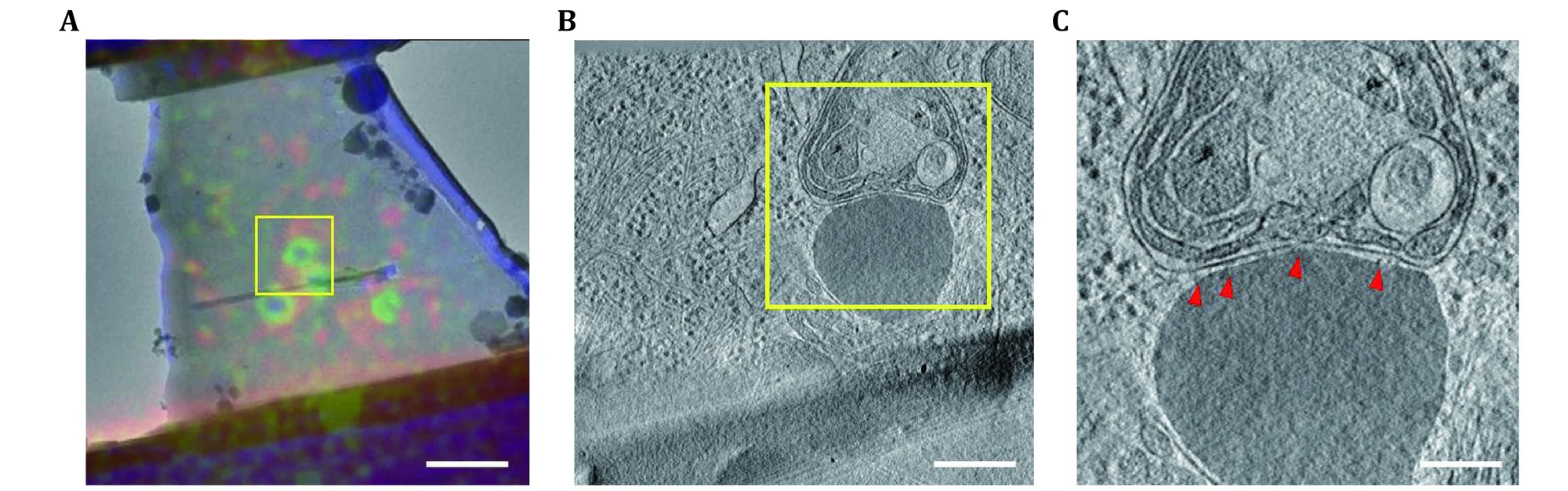
**A** ROI (yellow square) for Cryo-ET data collection, scale bar: 2 μm. **B** The tomogram of the ROI in Panel A, scale bar: 300 nm. **C** The detailed image of the yellow square in Panel B, showing the tethers (red arrows) at the contact site of LD and mitochondria, scale bar: 150 nm

4　Run the cryo-ET tilt series data collection procedure in the region of the interested target (yellow square in Fig. 10A).

**[NOTE]** Cryo-ET data collection should avoid crystalline regions. When using this plunge freezing protocol with PBS-diluted culture medium, the formation of crystalline areas is typically limited to less than 20%.

## RESULTS

In this study, we present a protocol for utilizing LM to facilitate site-specific FIB milling and to enhance the efficiency of cryo-ET data acquisition. A critical aspect of this approach is the acquisition of high-quality 3D LM images of vitrified cells. As demonstrated in Fig. 5, the 3D LM images allow for projection at arbitrary FIB milling angles, thereby enabling the alignment of LM and FIB images to the same viewing perspective (Fig. 6). Target localization is then achieved by measuring the relative position of the target to an internal FIB-milled benchmark (cross mark) in both imaging modalities. Furthermore, the integrated LM-FIB-SEM system enables on-the-fly fluorescence monitoring of the target during the lamella milling process, ensuring its presence throughout the procedure (Fig. 7). The final LM image of the milled lamella can be acquired to confirm the target's presence before initiating the cryo-ET experiment (Fig. 8). Additionally, the LM image of the final lamella significantly facilitates cryo-ET by correlating it with low-magnification TEM images (Fig. 9). This correlation greatly simplifies target identification on the lamella, a task that would otherwise be challenging due to the inherently low contrast of TEM images.

## DISCUSSION AND CONCLUSION

Traditional LM-guided lamella preparation using separate systems requires multiple transfers of vitrified samples between different instruments. This process often leads to ice contamination, sample devitrification, or sample damage. Conventional correlation methods rely on additional fiducial markers and coordinate transformations, which not only add complexity but also result in low accuracy. In this protocol, we introduce an improved approach for LM-guided lamella preparation using an integrated LM-FIB-SEM system. Performing both LM imaging and site-specific FIB milling within a single instrument significantly increases the efficiency and the success rate. Additionally, the correlation methodology based on LM image projection greatly enhances accuracy, while the use of an FIB-etched cross mark as a correlation benchmark eliminates the need for fiducial markers. Notably, the high-quality LM image of the final lamella, when correlated with TEM images, facilitates precise target localization for cryo-ET.

The throughput of site-specific lamella preparation using this protocol is approximately one hour per lamella, which is comparable to that of the most advanced integrated systems, such as the ELI trifocal microscope (Li *et al.* 2023a). This is significantly faster than other conventional CLEM systems, which typically require several hours to prepare a lamella at a specific site. The correlative milling accuracy achieved with this protocol is on the order of several tens of nanometers, enabling molecular-scale lamella preparation. This high precision extends applicability to a broader range of biological targets, including subcellular organelles, viruses, and macromolecular complexes. In contrast, other reported CLEM systems are generally limited to micrometer-scale targets, such as entire cells or certain subcellular regions, due to lower localization accuracy and limited fluorescence imaging quality.

The CLIEM system and this protocol can be readily adapted to other conventional FIB-SEM systems. On the hardware side, our integrated confocal microscope was designed as an add-on module that can be installed without requiring any modifications to the existing FIB-SEM hardware. On the software side, our correlation workflow is based on the open-source platform Fiji and its existing plugins, making it easily accessible and usable by other researchers.

Overall, the implementation of this integrated approach greatly enhances the throughput of the cryo-ET workflow, facilitating site-specific lamella preparation and precise target identification in TEM, thereby advancing the cryo-ET research.
